# *In vitro* susceptibility of *Escherichia coli* strains isolated from urine samples obtained in mainland China to fosfomycin trometamol and other antibiotics: a 9-year surveillance study (2004–2012)

**DOI:** 10.1186/1471-2334-14-66

**Published:** 2014-02-06

**Authors:** Bei Lai, Bo Zheng, Yun Li, Sainan Zhu, Zhaohui Tong

**Affiliations:** 1 Department of Respiratory and Critical Care Medicine, Beijing Institute of Respiratory Medicine, Beijing Chaoyang Hospital, Capital Medical University, Beijing 100020, PR of China; 2 Institute of Clinical Pharmacology, Peking University First Hospital, Beijing 100034, PR of China; 3 Department of Biostatistics, Peking University First Hospital, Beijing 100034, PR of China; 4 Department of Rheumatology and Immunology, Beijing Hosptial, Beijing 100730, PR of China

## Abstract

**Background:**

As a result of extensive use of fluroquinlones and cephalosporins, urinary tract pathogens producing extended-spectrum beta-lactamase (ESBL) pose a considerable clinical challenge in the treatment of UTIs. In the present study we retrospectively assessed the susceptibility of *E. coli* strains to fosfomycin trometamol and other antibiotics commonly used for the treatment of such infections.

**Methods:**

A total of 908 nonreplicate clinical *E. coli* urinary isolates were collected from 20 Chinese hospitals over four consecutive 1-year periods between October 2004 and June 2012. Susceptibility to antimicrobial agents fosfomycin trometamol, piperacillin-tazobactam, cefuroxime, cefotaxime, cefepime, imipenem, amikacin, levofloxacin, and nitrofurantoin was determined using the agar dilution method. A reference strain *E. coli* (ATCC 25922) was used as a positive control. Results were analyzed using Chi-square test or Fisher’s exact tests.

**Results:**

Fosfomycin trometamol, piperacillin-tazobactam, amikacin, and imipenem were consistently the most active agents against most of the isolates. There was a decline in susceptibility to cefuroxime, cefotaxime, and cefepime between 2004 and 2010. We showed that 528 of the 908 *E. coli* isolates (58.1%) produced ESBLs. The ESBL-positive rates increased from 41.7% in 2004−2005 to 60.9% in 2011−2012. ESBL-producing *E. coli* isolates showed significantly higher resistance rates to levofloxacin than the ESBL-negative isolates. Fosfomycin trometamol, piperacillin-tazobactam, amikacin, and imipenem had good activity against both levofloxacin-susceptible and levofloxacin- nonsusceptible isolates (sensitivity rate > 90%). However susceptibility of levofloxacin-resistant isolates to cefuroxime, cefotaxime, cefepime, amikacin, and nitrofurantoin was significantly lower than that of levofloxacin-susceptible isolates.

**Conclusions:**

Owing to the increase in the bacterial resistance across the world, the European Urology Association has recommended fosfomycin trometamol as the drug of choice in its Guidelines on Urological Infections released in 2013. Our results confirm this recommendation for use in China and continued monitoring of the susceptibility of *E. coli* to fosfomycin trometamol is need with the widely use of the drug in China.

## Background

Urinary tract infections (UTIs) are among the most common conditions, which require diagnostic and therapeutic intervention. *Escherichia coli* is to date the most frequent uropathogen, which accounted for 30.7% and 51.5% of all UTIs in men and women, respectively, during 2010 in China [1]. Fluoroquinolones and cephalosporins were the most commonly used antibiotics for the treatment of UTIs in China. However, in the recent years, many resistant strains have emerged because of the use of broad-spectrum cephalosporins. In particular, strains producing extended-spectrum beta-lactamase (ESBL) pose a considerable clinical challenge in the treatment of UTIs.

Fosfomycin has been extensively used in several European countries for the treatment of uncomplicated UTIs since 1988, but it was not available in China until recently. This study aimed to reassess the susceptibility of *E. coli* strains isolated from patients with UTIs between October 2004 and June 2012 to fosfomycin trometamol and other antibiotics commonly used for the treatment of such infections.

## Methods

### Bacterial isolates

A total of 908 nonreplicate clinical *E. coli* urinary isolates were collected from 20 widely dispersed tertiary Chinese hospitals over four 1-year periods (October 2004–September 2005, January 2007–December 2007, July 2009–June 2010, and July 2011–June 2012) and were sent to the Institute of Clinical Pharmacology, Peking University First Hospital; the isolates were stored at −80°C until further analysis. The isolates were stored at −80°C until further analysis. The strains collected from 20 widely dispersed tertiary Chinese hospitals participating in the Ministry of Health National Antimicrobial Resistance Surveillance Net study. These hospitals are located in 15 different provinces in mainland China. The number of isolates from per hospital per year was from 4 to 26.

### Antimicrobial susceptibility testing

Susceptibility to antimicrobial agents was determined using the agar dilution method according to the Clinical and Laboratory Standards Institute [2]. The following antimicrobial agents were tested: fosfomycin trometamol, piperacillin-tazobactam, cefuroxime, cefotaxime, cefepime, imipenem, amikacin, levofloxacin, and nitrofurantoin. ESBL)-producing isolates were detected according to previously described methods [2]. The reference strain *E. coli* ATCC 25922 was used as the positive control.

### Statistical analyses

Statistical tests were performed using Social Sciences software for Windows Version 14.0 (SPSS, Inc., Chicago, IL, USA). Enumeration data were expressed as percentage values. The differences in susceptibility between the groups were compared using the Chi-square test or Fisher’s exact test. The differences between the groups were considered significant if the p-values were smaller than 0.05(two-sided test). The Bonferroni method was used to adjust the significant levels (0.05/6 = 0.0083) in multiple comparisons between any two levels of the susceptibility outcome.

## Results

### Comparison of antimicrobial susceptibilities between 2004 and 2012

Fosfomycin trometamol, piperacillin-tazobactam, amikacin, and imipenem were consistently the most active agents against most of the isolates. We observed a decline in the susceptibility to cefuroxime, cefotaxime, and cefepime from 2004 to 2010. In particular, the susceptibility to cefepime decreased significantly from 88.1% during October 2004–September 2005 to 61.5% during July 2009–June 2010 (p < 0.0001). Over the same period, the minimum inhibitory concentration inhibiting 90% of the strains (MIC_90_) increased from 16 mg/L to 64 mg/L. Susceptibility to nitrofurantoin was 78.6% during October 2004–September 2005, which increased to 83.0% during January 2007–December 2007 and to 91.6% during July 2009–June 2010 period (p < 0.0001) (Table 1).

**Table 1:** Comparison of antimicrobial susceptibilities between 2004 and 2012

**Antibiotic**^**a**^	**10/2004–9/2005 (*n*** **= 168)**		**1/2007–12/2007 (*n*** **= 271)**		**7/2009–6/2010 (*n*** **= 262)**		**7/2011–6/2012 (*n*** **= 207)**		**p**
	**S (%)**	**MIC**_**90 **_**(mg/L)**	**S (%)**	**MIC**_**90 **_**(mg/L)**	**S (%)**	**MIC**_**90 **_**(mg/L)**	**S (%)**	**MIC**_**90 **_**(mg/L)**	
FOS	99.4	2	95.9	1	95.0	4	93.2*	8	0.030
TZP	94.0	16	97.8	8	92.7	16	94.7	8	0.058
CXM	42.3	512	32.8	512	27.5*	512	30.9	512	0.015
CTX	53.0	128	37.6*	256	30.9*	512	36.2*	512	< 0.001
FEP	88.1	16	76.4*	32	61.5*^,^**	64	75.4*^,^***	32	< 0.001
IMP	100	0.25	99.6	0.25	99.6	0.125	99.5	0.125	1.000
AMK	94.6	4	95.6	4	92.7	8	93.2	4	0.519
NIT	78.6	64	83.0	64	91.6*^,^**	32	88.9*	64	< 0.001
LVX	31.0	32	28.0	32	28.2	32	32.4	32	0.693

^a^Drug abbreviations: FOS, fosfomycin trometamol; TZP, piperacillin-tazobactam; CXM, cefuroxime; CTX, cefotaxime; FEP, cefepime; IMP, imipenem; AMK, amikacin; NIT, nitrofurantoin; LVX, levofloxacin.

*compared to 10/2004–9/2005, p < 0.0083.

**compared to 1/2007–12/2007, p < 0.0083.

***compared to 7/2009–6/2010, p < 0.0083.

### Comparison of antimicrobial susceptibilities to ESBL-producing strains of *E. coli*

In this study, 528 *E. coli* isolates (58.1%) produced ESBLs. The ESBL-positive rates were 41.7% (70/168), 58.7% (159/271), 66.0% (173/262), and 60.9% (126/207) in the four periods from 2004 to 2012, respectively. ESBL-producing *E. coli* isolates showed significantly higher resistance rates to cefepime and levofloxacin than the ESBL-negative isolates. Fosfomycin trometamol, piperacillin-tazobactam, imipenem, amikacin, and nitrofurantoin showed good activity against both ESBL-producing and ESB-negative isolates; the sensitivity rates were greater than 85% (Table 2).

**Table 2:** **Comparison of the susceptibilities of ESBL-producing *Escherichia coli *** **strains to various antimicrobial agents**

**Antibiotic**	**ESBL-positive (*n*** **= 528)**		**ESBL-negative (*n*** **= 380)**		**p**
	**S (%)**	**MIC**_**90 **_**(mg/L)**	**S (%)**	**MIC**_**90 **_**(mg/L)**	
FOS	93.8	2	98.4	1	0.001
TZP	95.3	16	95.8	8	0.707
IMP	100	0.25	99.2	0.25	0.073
AMK	92.8	8	95.8	4	0.061
NIT	86.2	64	85.8	64	0.869
LVX	17.2	32	46.8	32	< 0.001

^a^Drug abbreviations: FOS, fosfomycin trometamol; TZP, piperacillin-tazobactam; IMP, imipenem; AMK, amikacin; NIT, nitrofurantoin; LVX, levofloxacin; ESBL, extended-spectrum beta-lactamase.

### Comparison of susceptibility of levofloxacin-resistant and levofloxacin-susceptible strains of *E. coli* to various antimicrobial agents

Fosfomycin trometamol, piperacillin-tazobactam, amikacin, and imipenem had good activity against both levofloxacin-susceptible and levofloxacin- nonsusceptible isolates (sensitivity rate > 90%). The rate of susceptibility to cefuroxime, cefotaxime, cefepime, amikacin, and nitrofurantoin was significantly lower in the levofloxacin-resistant isolates than in the levofloxacin-susceptible isolates (p < 0.001) (Table 3).

**Table 3:** **Comparison of susceptibility of levofloxacin-susceptible and levofloxacin- nonsusceptible strains of *Escherichia coli *** **to various antimicrobial agents**

**Antibiotic**^**a**^	**LVX-S (*n*** **= 269)**		**LVX-NS (*n*** **= 639)**		**p**
	**S (%)**	**MIC**_**90 **_**(mg/L)**	**S (%)**	**MIC**_**90 **_**(mg/L)**	
FOS	98.5	0.5	94.5	4	0.007
TZP	95.9	4	95.3	8	0.688
CXM	63.2	512	19.7	512	< 0.001
CTX	64.7	128	27.1	512	< 0.001
FEP	91.4	8	66.7	64	< 0.001
IMP	100	0.25	99.5	0.25	0.559
AMK	98.9	2	92.0	8	< 0.001
NIT	96.3	16	81.7	64	< 0.001

LVX-S, levofloxacin-susceptible; LVX-NS, levofloxacin-resistant or intermediately.

^a^Drug abbreviations: FOS, fosfomycin trometamol; TZP, piperacillin-tazobactam; CXM, cefuroxime; CTX, cefotaxime; FEP, cefepime; IMP, imipenem; AMK, amikacin; NIT, nitrofurantoin; LVX, levofloxacin.

## Discussion and conclusion

*E. coli* plays an important role in UTI; UTI is one of the most frequently encountered infectious diseases. Cephalosporins and levofloxacin are the widely used agents for the treatment of UTI. The production of ESBLs by *E. coli* is associated with the reduced susceptibility to fluoroquinolones and other antimicrobial agents. The resistance rates of ESBL-producing *E. coli* to levofloxacin in this study were higher than those reported in other studies performed in different countries [3,4]. Nitrofurantoin were seldom used in China, it is maybe the reason that the susceptibility of nitrofurantoin increased over time.

Our data confirm that *E. coli* isolated from Chinese UTI patients remain exclusively susceptible to fosfomycin trometamol. In addition, the ESBL-producing and levofloxacin-resistant strains of *E. coli* were susceptible to fosfomycin trometamol. Our results are similar to those reported in recent studies on the same pathogens isolated from other areas of the world [5,6].

Fosfomycin trometamol was excreted renally within 48 hours and urinary titres showed that concentrations > 128 mg/L, sufficient to inhibit most urinary pathogens, were maintained for 24 to 48 hours after a single oral dose (≅3g fosfomycin), but the mean peak plasma concentrations were only 22 to 32 mg/L [7]The European Urology Association has recommended fosfomycin trometamol, nitrofurantoin and pivmecillinam as the drug of choice in its Guidelines on Urological Infections released in 2013 [8]. An uncontrolled and multicentre study showed clinical efficacy rates for acute uncomplicated cystitis, recurrent lower urinary tract infection and complicated lower urinary tract infection were 94.71, 77.22% and 62.69% [9]. Continued monitoring of the susceptibility of *E.coli* to fosfomycin trometamol is needed with the widely use of the drug in China.

## Competing interests

The authors declare that they have no conflict of interest.

## Authors’ contributions

BL, BZ and YL carried out the antimicrobial susceptibility testing and drafted the manuscript. SZ participated in the design of the study and performed the statistical analysis. ZT conceived of the study, and participated in its design and coordination and helped to draft the manuscript. All authors read and approved the final manuscript.

## Pre-publication history

The pre-publication history for this paper can be accessed here:

http://www.biomedcentral.com/1471-2334/14/66/prepub
